# Transvaginal ultrasound diagnosis of a rare entity: Premenopausal ovarian hyperthecosis

**DOI:** 10.1002/ccr3.9380

**Published:** 2024-08-28

**Authors:** Benedetta Cornelli, Wouter Froyman, Giulia Garofalo

**Affiliations:** ^1^ Department of Obstetrics and Gynaecology, CHU Saint Pierre Université Libre de Bruxelles Bruxelles Belgium; ^2^ Department of Development and Regeneration KU Leuven Leuven Belgium; ^3^ Department of Obstetrics and Gynaecology University Hospitals Leuven Leuven Belgium; ^4^ HUB, Hôpital Erasme, Université Libre de Bruxelles Bruxelles Belgium

**Keywords:** ovarian cysts, ovary, premenopause, theca cells, ultrasonography

## Abstract

Ovarian hyperthecosis (OH) is a benign pathology, less common in premenopause. Literature is poor on its ultrasound (US) characteristics. We suggest that a heterogeneous ovary at US, with a central vascularisation and follicles to the periphery, with or without hyperandrogenism, should lead to consider OH in the hands of experts.

## Graphical abstract

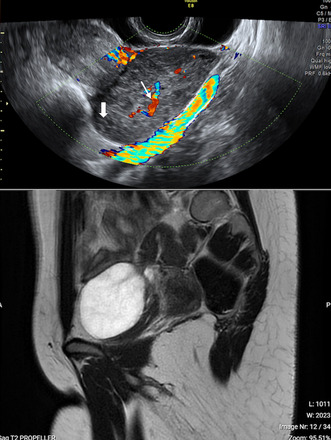

## 1 INTRODUCTION

Ovarian hyperthecosis (OH) is a non‐neoplastic pathology of the ovaries in which follicles have luteinized theca cells and are scattered in a generally hyperplastic stroma. This determines an excess of androgen production and can result in a wide spectrum of clinical presentations.^1^, ^2^ OH is more frequent in postmenopausal women, where it tends to have a mild or a null clinical significance. However, few cases of virilization or worsening of previously existing symptoms of androgen excess have been reported.^3^, ^4^ On the contrary, in premenopausal women, OH is less frequent but usually associated with major virilization symptoms such as acne, seborrhoea, hirsutism, temporal balding, obesity, hypertension, or even impaired glucose tolerance.^5^

Even though hyperandrogenism is a common indication for ultrasound (US), literature on US characteristics of OH, specifically in the latter subgroup of patients, is scarce. It has been reported that the ovaries can appear totally normal, mildly enlarged but otherwise normal, or with an increased stroma that gently pushes normal follicles towards the periphery.^3^, ^6^

We present hereby two cases of ultrasonographic diagnosis of OH in young asymptomatic women with a pelvic mass.

## 2 CASE HISTORY 1

A 27‐year‐old nulliparous woman was addressed to our gynecology department, due to the suspicion of a dermoid cyst in the right ovary, discovered accidentally.

She used to take oral oestro‐progestinic contraception since adolescence for facial acne with reported biological hyperandrogenism, but switched to oral progestins (OP) with desogestrel after the diagnosis of a heterozygous factor V Leiden mutation.

Her general and gynecological examination was unremarkable, and she presented a modified Ferriman‐Gallwey score (FGS) of 4/36 (after laser therapy on the legs).

### 2.1 Methods

**FIGURE 1:**
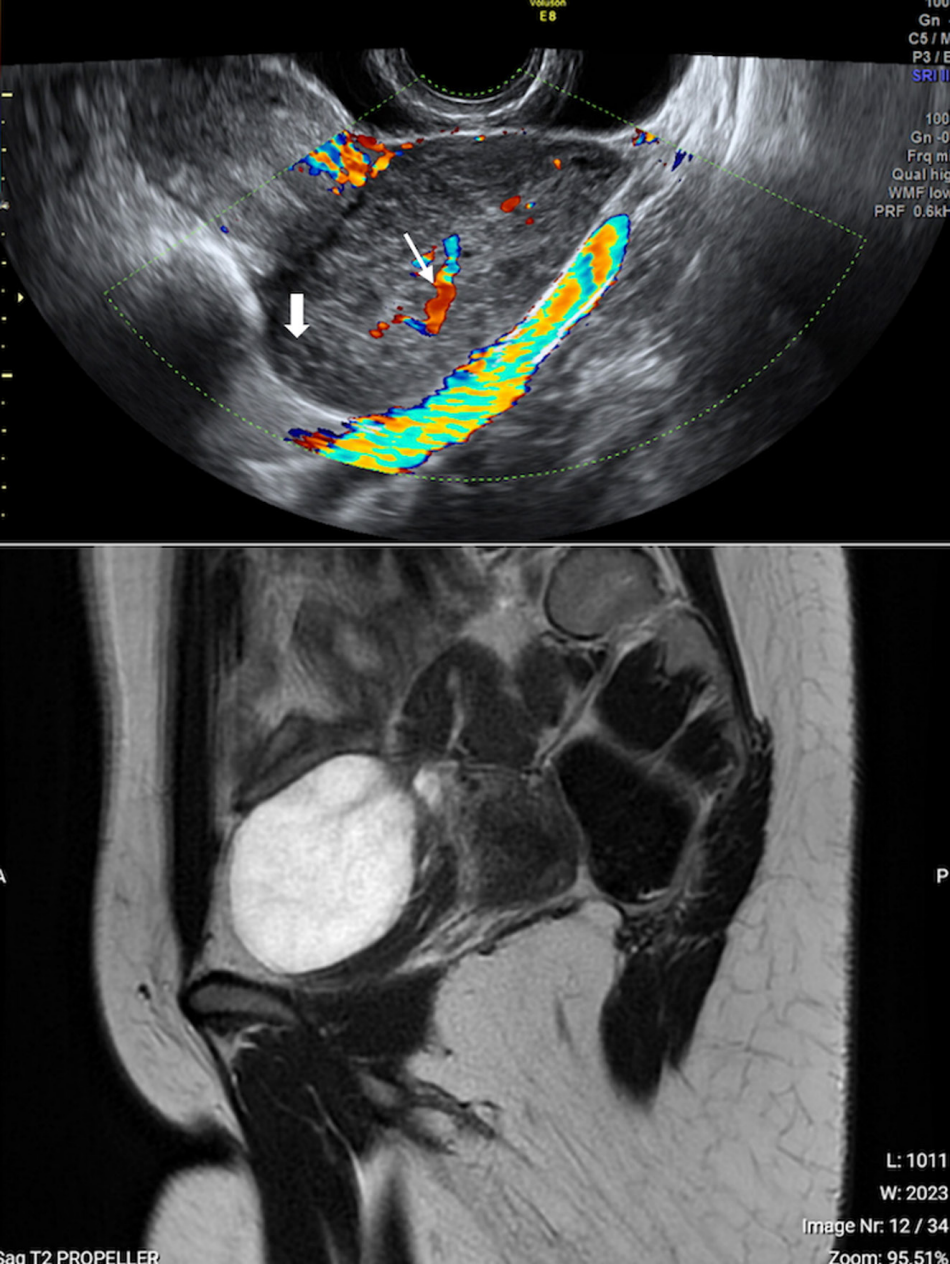
Ultrasound pattern of case 1 (heterogeneous parenchyma without acoustic shadows, with follicles in the periphery, thick arrow, and a central vascular axis color score 2, thin arrow) and corresponding magnetic resonance imaging.

On transvaginal US the right ovary was enlarged measuring 71 × 35 × 68 mm. The whole parenchyma showed an heterogeneous pattern without acoustic shadows, with follicles being mainly in the periphery and a central vascular axis color score 2 (Figure 1). No distinct mass was visible. The left ovary and the endometrium were normal.

**FIGURE 2:**
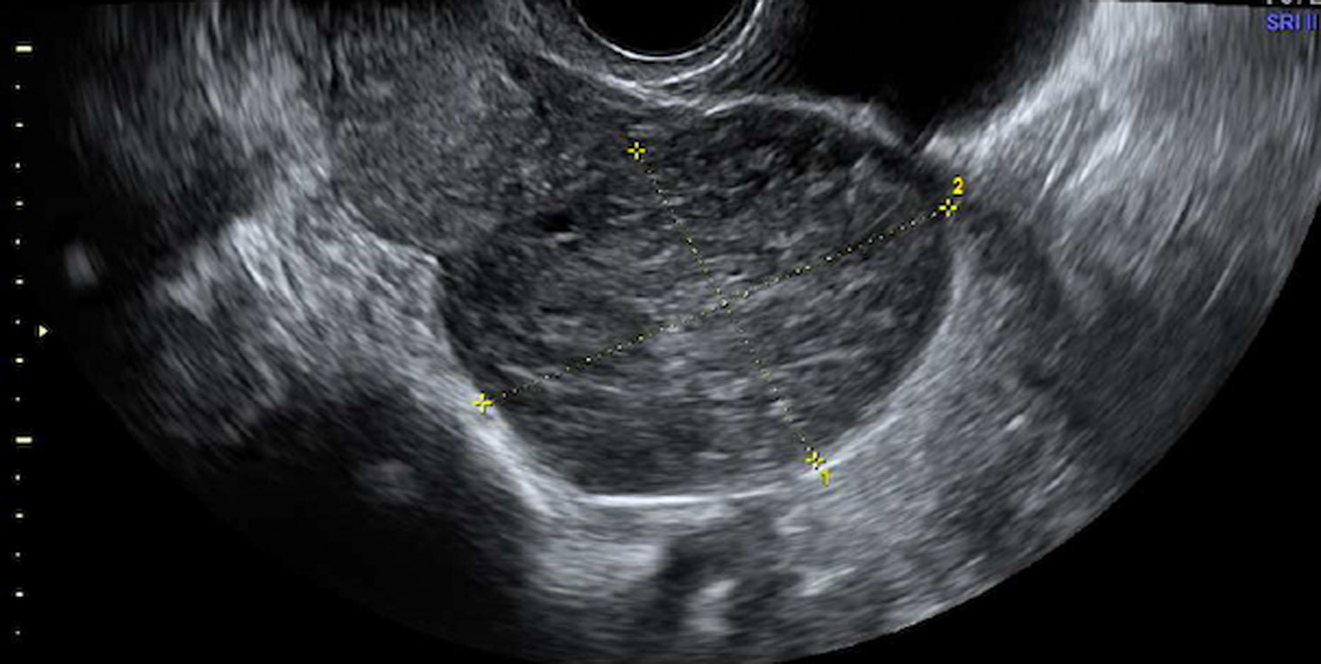
Maximum diameter at first diagnosis at ultrasound of case 1.

The suspect of stromal OH was posed. Applying the Assessment of Different NEoplasias (ADNEX) model though,^7^ the absolute risk of malignancy was 50.6%. The highest absolute subgroup risk of malignancy was ovarian cancer stage II–IV (21.8%) and the highest subgroup relative risk (RR) of malignancy was of 2.28 for metastasis to the adnexa. A magnetic resonance imaging (MRI) was offered and it confirmed the suspicion of OH (Figures 1 and 2). Of note, the patient underwent an abdominal computed tomography (CT) scan 3 months before the US and it showed a cystic lesion of 63 × 48 mm on the right ovary, with few thin septa, without solid tissue.

### 2.2 Conclusions and results

A clinical and US follow up at 3 months was offered: this showed a stable US aspect and a small dimension reduction (58 × 31 × 58 mm), authorizing the following control at 6 months. Afterwards the patient had decided to stop the OP due to persistent spotting.

**FIGURE 3:**
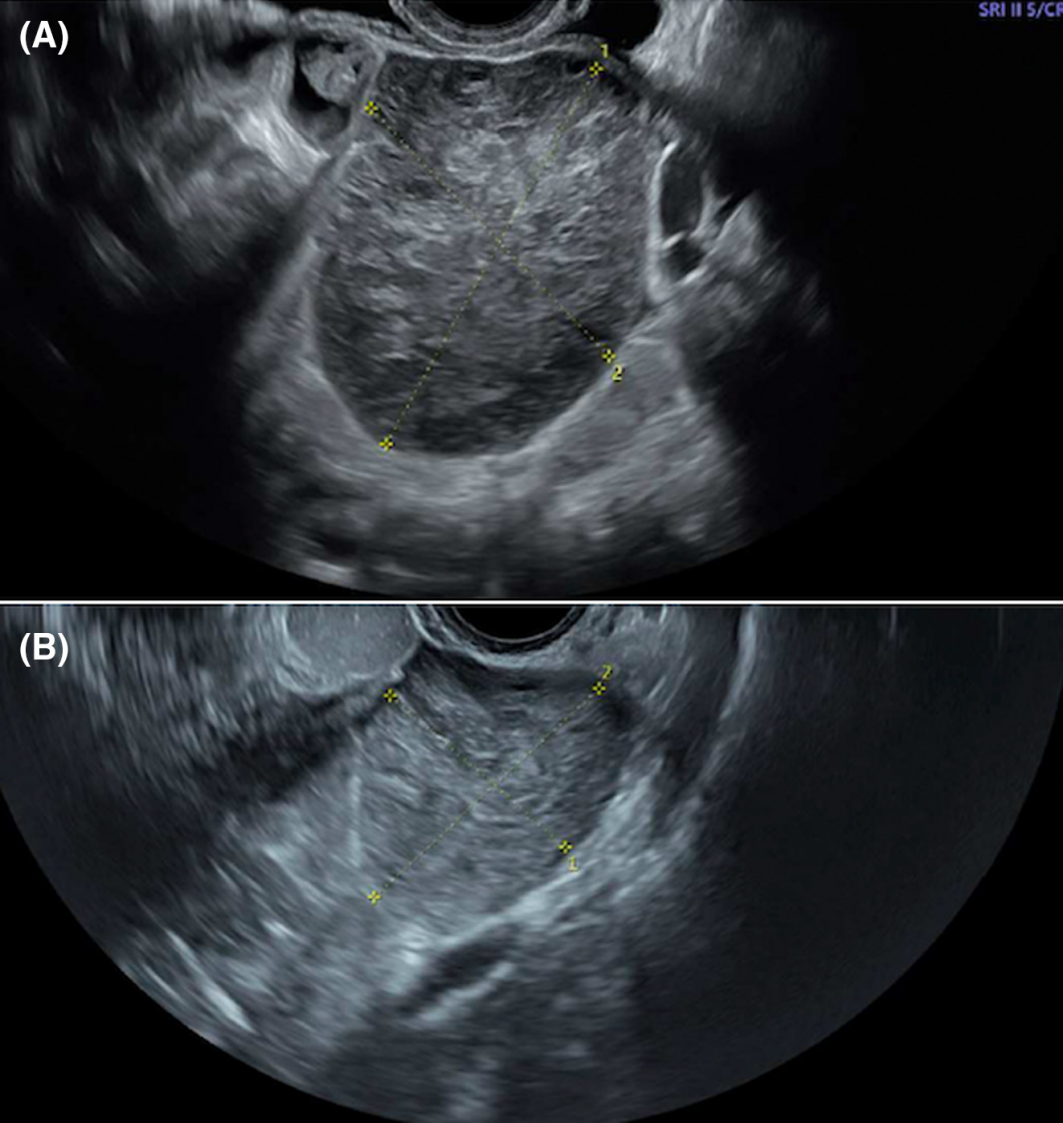
Dimensions at ultrasound of case 1 before (A) and after (B) treatment with drospirenone.

In the following US, the lesion showed the same US aspect but the dimensions increased, to 75 × 60 × 78 mm (Figure 3A). The contralateral ovary had for the first time a micropolycystic (PCO) aspect. Her period was regular of 28 days but the patient started to have slight abdominal noncontinuous pain.

A hormonal evaluation in the 3rd day of period showed an elevated total testosterone with a normal free component (total 2.24 nmol/L [normal 0.40–1.67], free 19.2 pmol/L [normal 3.0–37.0]), an elevated androstanediol glucuronide (22.6 nmol/L [normal 1.0–16.0]), and a normal delta 4 androstenedione (Delta 4 A) (1.9 ng/mL [normal 0.1–3.0]). Dehydroepiandrosterone sulfate (DHEA‐S) was slightly elevated (12.10 μmol/L [normal 2.68–9.23]), as it was 17‐hydroxyprogesterone (4.6 ng/mL [normal 0.4–1.5]). Gonadotropins, oestradiol, and SHBG were in the range. A mild hyperprolactinemia was noted (total 45.9 μg/L [normal 4.8–23.3], bioactive 37.7 μg/L [normal 3.5–18]).

We proposed then to start an OP with drospirenone.

At last ultrasound, 6 months after, the right ovarian dimensions were reduced (42 × 54 × 59 mm) (Figure 3B), but the left ovary appeared to have increased in volume (54 × 62 × 46 mm) and it showed for the first time a similar US features to the right one.

The stability of the US pattern and the good tolerance of the medical therapy allowed us to propose yearly follow up.

## 3 CASE HISTORY 2

A 22‐year‐old virgo woman was addressed to our gynaecologic department for a right‐sided ovarian mass discovered after emergency unit access 2 weeks before for acute right iliac fossa pain without any other symptoms. She was discharged with outpatient follow up at the time after pain relief with intravenous paracetamol and tramadol. Her medical history was unremarkable.

### 3.1 Methods

**FIGURE 4:**
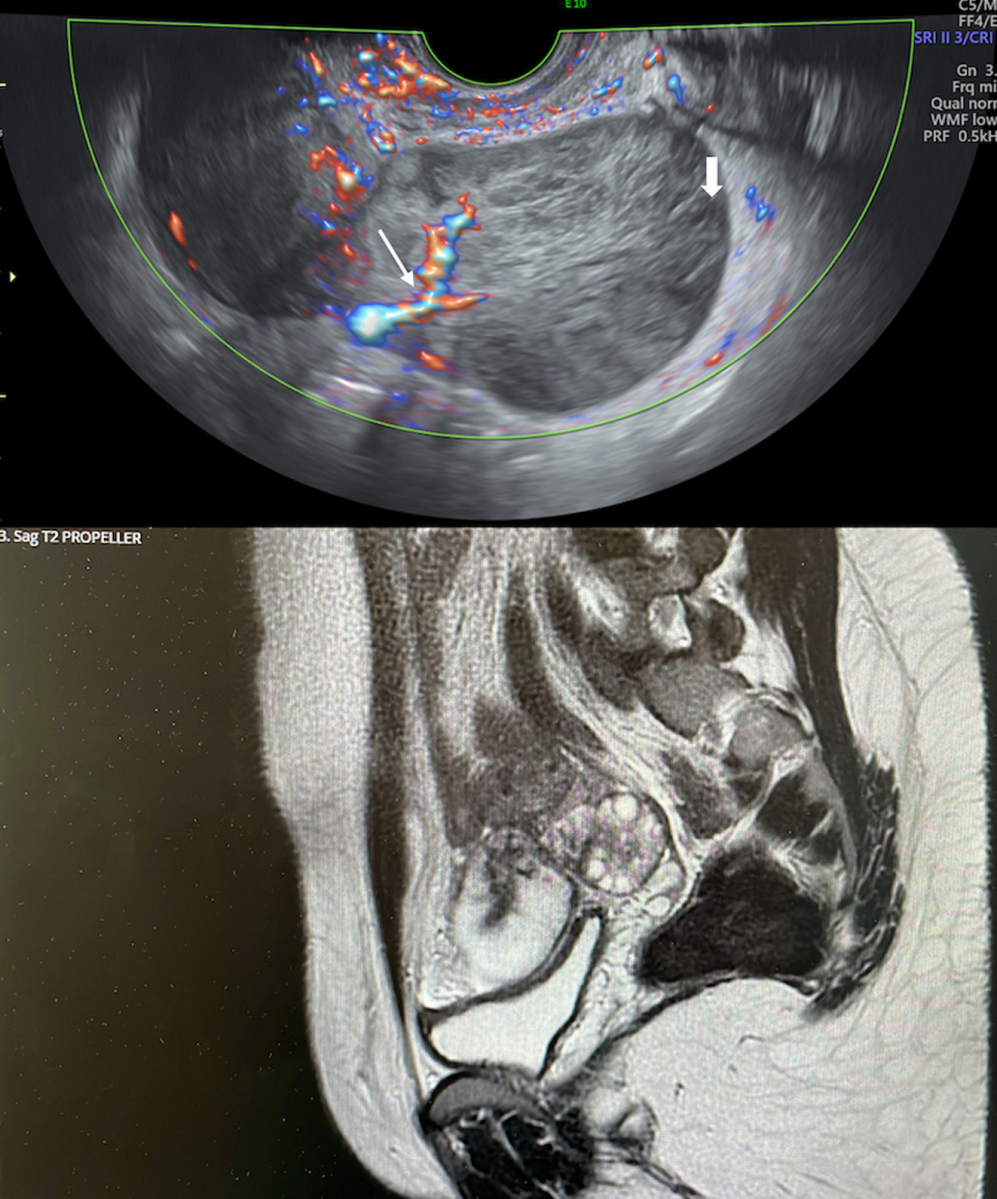
Ultrasound pattern of case 2 (heterogeneous parenchyma without acoustic shadows, with follicles in the periphery, thick arrow, and a central vascular axis color score 2, thin arrow) and corresponding magnetic resonance imaging (enlarged ovary with haemorrhagic component).

**FIGURE 5:**
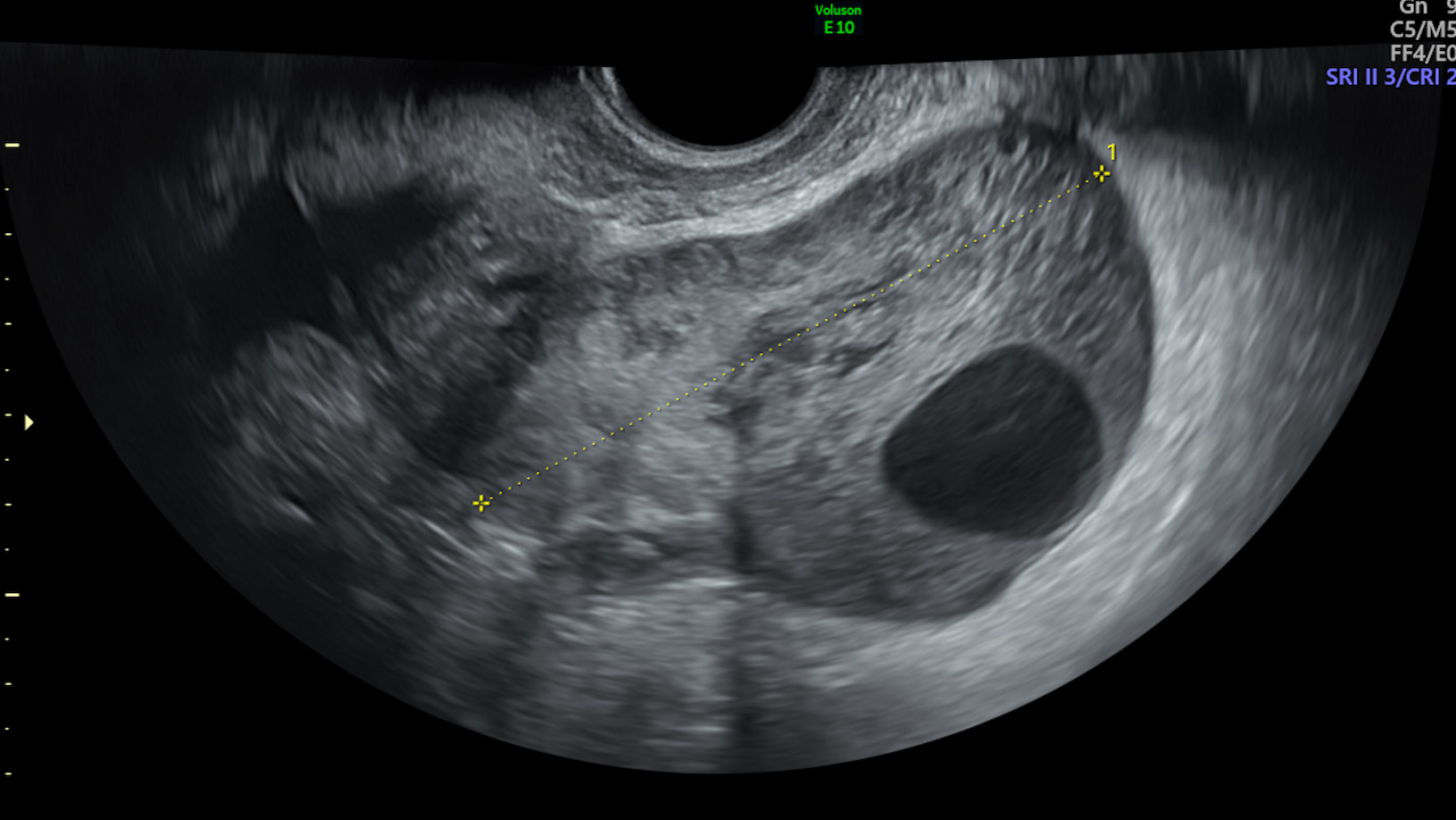
Maximum diameter at ultrasound of case 2.

On transrectal US scan, the right ovary appeared enlarged (93 × 53 × 78 mm) without a well‐defined mass, but showed a heterogeneous aspect of the parenchyma and a peripheral follicular distribution. We visualized a central vascular axis with thin branches, color score 3 (Figures 4 and 5). The left ovary instead presented a PCO aspect and measured 45 × 47 × 25 mm. The uterus appeared normal. At the time of the US the patient was asymptomatic and an OH was suspected.

The ADNEX model, again, suggested an absolute risk of malignancy of 54.2% for which the highest absolute risk of malignancy was of 21.6% for ovarian cancer stage II–I. The highest subgroup RR of malignancy was de 2.45 for ovarian cancer stage I. An MRI was performed a week after the US and confirmed an enlarged right ovary (85 x 45 mm in the axial plane) with haemorrhagic component (Figure 2).

### 3.2 Conclusions and results

Giving the suspicion of OH, an OP treatment with drospirenone was proposed to this patient.

After less than 2 months with OP treatment, the US scan revealed a normal right ovary and normal sized left ovary that kept his PCO US aspect.

Treatment was continued and a yearly follow up was proposed.

## 4 DISCUSSION

OH is less frequent in premenopausal women but it usually shows a more severe phenotype with virilization symptoms, obesity, hypertension, and impaired glucose tolerance. However, its clinical impact can vary a lot, being less evident in adolescence. Moreover, symptoms of hyperandrogenism may resemble those of polycystic ovary syndrome (PCOS), and it has been suggested that OH and PCOS could be two different manifestations of the same heterogeneous dysfunction of androgens metabolism.^8^, ^9^

However, in our cases, hyperandrogenism was not the indication of the US. In our first patient, we could note an anamnestic clinical and biological hyperandrogenism. In the recent blood test, total testosterone, androstenediol glucuronide, and DHEAS resulted mildly elevated. Moreover, the FGS was not significant for the diagnosis of hirsutism but the patient had a previous laser therapy, and this treatment is often due to excessively dark, thick, or dense hairs. Notwithstanding, we could consider her current clinical and biological phenotype nearly normal. In the second patient, there was no medical history nor clinical sign of hyperandrogenism. No blood tests were performed due to the fast resolution under OP.

Due to the rarity of OH in premenopausal women especially when pauci‐symptomatic, it is understandable that the literature is quite poor regarding the US features of ovaries with OH in these patients.

To our knowledge, only two case series focused on the US characteristics of histologically proven OH in premenopausal women. Brown et al^6^ collected the transvaginal or transabdominal US of 14 women. They concluded that the most frequent findings were a normal ovary or a mildly enlarged but otherwise normal ovary, and six of these patients showed a unilateral involvement. We can reasonably consider four patients as premenopausal because they are younger than 45 years. Two of these latter were asymptomatic. Two of these patients had a unilaterally enlarged ovary, with a normal appearance. One patient presented with a cyst described as “haemorrhagic” of 35 mm with “mild” OH at the histology. In the last patient both ovaries were enlarged with PCO‐like appearance: the ovarian histology showed OH and PCO.

In the Rousset et al.^3^ case series, two out of 10 women were premenopausal, and ovaries were always bilaterally affected. The main US pattern showed enlarged but otherwise normal ovaries. Doppler was used for six patients and it showed a homogeneous centrally located color flow in four patients and no signal in two women. The two premenopausal women were 18 and 30 years old, respectively, and showed clinical and biological hyperandrogenism. For one patient, US revealed an increased stroma with peripheral follicles and both patients presented a doppler flow with the pattern described above.

In both our cases, the contralateral ovary showed a PCO‐like appearance, supporting a possible correlation between these two entities. An interesting finding is the “haemorrhagic” cyst in one premenopausal patient of Brown's report and the haemorrhagic aspect in the MRI of our second patient. Moreover, the central vascular axis and follicles confined to the periphery was crucial in both cases for the suspicion of OH, also in line a case of the Rousset's study.

The MRI was mandatory to confirm our suspicion, even if literature is scarce also for these features. However, the MRI pattern reported to be suspicious of OH is symmetric bilateral ovarian enlargement with central hypointensity on T1‐weighted images and hyperintensity on T2‐weighted images and isointensity relative to myometrium on both T1‐ and T2‐weighted imaging in the peripheries.^10^

Of note is the risk assessed by the Adnex Model of the IOTA group^7^ in both our cases: since it does not apply to cases without a proper mass, when wrongly used as in the two cases reported, it suggested a malignant lesion. However, the expert opinion of two different imaging techniques helped us to rule out the presence of a solid lesion inside the ovary in favor of an enlarged ovary with a particular pattern.

Considering this, due to the young age, the nulliparity and compliance to follow‐up of our patients, we decided to propose conservative treatment rather than a surgical one and thus sparing their fertility. The stability of the US appearance was reassuring, reinforcing the hypothesis of a benign lesion. The longest follow up has been 24 months so far, for the first patient.

The slight shrinking of ovarian dimensions in the first patient and the complete resolution of the ovarian features in the second one under oral drospirenone was very remarkable. The choice of this specific OP was tailored by the US findings, and to our knowledge, there is no description of such findings in the literature.

The frequent use of transvaginal US and the technical improvement will probably lead to a rise in cases of US diagnosis of OH in premenopausal young women. It would be very interesting to compare these cases with other similar cases including their clinical and biological examination and their eventual response to antiandrogenic progestins.

The limit of our study is the absence of a histological confirmation. Moreover, two differential diagnoses (DD) have to be taken into account: ovarian torsion and the presence of a Sertoli‐Leydig cell tumor. Ovarian torsion could be suspected in the second case; however, the clinical symptoms were mild and the ovarian dimensions on US were too big to justify an ovarian torsion of a normal ovary. Moreover, no “whirpool sign”^11^ was observed. For the second DD, Sertoli‐Leydig cell tumors are usually reported in premenopausal women and up to 80% of them present with endocrine symptoms. They are mainly described as small or medium‐sized solid lesions or multilocular‐solid ovarian lesions of any size (3–18 cm), moderately or abundantly vascularized.^12^ The absence of clinical symptoms and a distinct ovarian mass on ultrasound were the main reasons to discard this hypothesis.

We can conclude by suggesting that the presence at transvaginal/transrectal US of a heterogeneous ovary as a whole, unilaterally or bilaterally, with a central vascular axis and follicles confined to the periphery in a premenopausal young woman, with or without hyperandrogenism, should lead to consider the diagnosis of OH in the hands of experienced sonographers.

## AUTHOR CONTRIBUTIONS

**Benedetta Cornelli:** Conceptualization; data curation; investigation; resources; validation; writing – original draft. **Wouter Froyman:** Data curation; investigation; methodology; resources; supervision; validation. **Giulia Garofalo:** Conceptualization; data curation; investigation; methodology; resources; supervision; validation; writing – original draft.

## CONFLICT OF INTEREST STATEMENT

Authors have nothing to disclose.

## CONSENT

Written informed consent was obtained from the patient to publish this report in accordance with the journal's patient consent policy.

## Data Availability

Data available on request from the authors.
